# Simple *In Vitro* Assay To Evaluate the Incorporation Efficiency of Ribonucleotide Analog 5′-Triphosphates into RNA by Human Mitochondrial DNA-Dependent RNA Polymerase

**DOI:** 10.1128/AAC.01830-17

**Published:** 2018-01-25

**Authors:** Gaofei Lu, Gregory R. Bluemling, Shuli Mao, Michael Hager, Bharat P. Gurale, Paul Collop, Damien Kuiper, Kasinath Sana, George R. Painter, Abel De La Rosa, Alexander A. Kolykhalov

**Affiliations:** a Emory Institute for Drug Development (EIDD), Emory University, Atlanta, Georgia, USA; b Drug Innovation Ventures at Emory (DRIVE), Atlanta, Georgia, USA; c Department of Pharmacology, Emory University School of Medicine, Atlanta, Georgia, USA

**Keywords:** discrimination value, POLRMT, RNA-dependent RNA polymerase, RdRp, human mitochondrial RNA polymerase, mitochondrial polymerase, nonradioactive assay, nucleoside analogs, primer extension, ribonucleotide

## Abstract

There is a growing body of evidence suggesting that some ribonucleoside/ribonucleotide analogs may be incorporated into mitochondrial RNA by human mitochondrial DNA-dependent RNA polymerase (POLRMT) and disrupt mitochondrial RNA synthesis. An assessment of the incorporation efficiency of a ribonucleotide analog 5′-triphosphate by POLRMT may be used to evaluate the potential mitochondrial toxicity of the analog early in the development process. In this report, we provide a simple method to prepare active recombinant POLRMT. A robust *in vitro* nonradioactive primer extension assay was developed to assay the incorporation efficiency of ribonucleotide analog 5′-triphosphates. Our results show that many ribonucleotide analogs, including some antiviral compounds currently in various preclinical or clinical development stages, can be incorporated into newly synthesized RNA by POLRMT and that the incorporation of some of them can lead to chain termination. The discrimination (*D*) values of ribonucleotide analog 5′-triphosphates over those of natural ribonucleotide triphosphates (rNTPs) were measured to evaluate the incorporation efficiency of the ribonucleotide analog 5′-triphosphates by POLRMT. The discrimination values of natural rNTPs under the condition of misincorporation by POLRMT were used as a reference to evaluate the potential mitochondrial toxicity of ribonucleotide analogs. We propose the following criteria for the potential mitochondrial toxicity of ribonucleotide analogs based on *D* values: a safe compound has a *D* value of >10^5^; a potentially toxic compound has a *D* value of >10^4^ but <10^5^; and a toxic compound has a *D* value of <10^4^. This report provides a simple screening method that should assist investigators in designing ribonucleoside-based drugs having lower mitochondrial toxicity.

## INTRODUCTION

Ribonucleotide analog 5′-triphosphates (the active form of ribonucleoside analogs) can compete with natural ribonucleotides for incorporation into elongating viral RNA that may disrupt viral RNA synthesis and, thus, inhibit viral replication. The nucleoside-based inhibitors (NIs) and ribonucleoside-based inhibitors (rNIs) targeting viral RNA polymerases have been proven to be the backbone of antiviral therapies (1). In recent years, they have been intensely studied as part of the fight against diseases caused by RNA viruses and have been particularly successful against hepatitis C virus (HCV) infection (2–4). The clinical applications of some new inhibitors have been hampered by the development of signs of toxicity. Toxicity issues associated with nucleoside-based drugs have been particularly challenging for long-term treatments. For example, for anti-HCV nucleotide inhibitors, only sofosbuvir has been approved by the FDA for the treatment of HCV infection, while the clinical development of many other ribonucleoside/ribonucleotide analogs, such as NM283 (a prodrug of 2′-C-Me-cytidine), RG1626 (a prodrug of 4′-azido-cytidine), PSI-938 and PSI-661 (prodrugs of 2′-F-2′-C-Me-guanosine), and BMS-986094 and IDX-184 (prodrugs of 2′-C-Me-guanosine), was terminated due to various toxicity issues (2). The failure of ribonucleoside/ribonucleotide analogs during clinical development has been due to toxicity that was not detected during preclinical studies. More effective drug screening methods that can be performed earlier in the drug development process are still needed to help identify the potential toxicity of rNIs before committing to their extensive preclinical and clinical development.

The toxicity associated with rNIs has not been well characterized. Since ribonucleotide analog 5′-triphosphates resemble natural ribonucleotide 5′-triphosphates (rNTPs), they theoretically could be incorporated into cellular RNA by human RNA polymerases I, II, and III (Pol I, Pol II, and Pol III, respectively) as well as by human mitochondrial DNA-dependent RNA polymerase (POLRMT). RNA polymerases I, II, and III possess strong intrinsic 3′ → 5′ nuclease activity (a proofreading activity), which can remove misincorporated ribonucleotides during transcription (5). It has been shown that Pol II can quantitatively remove misincorporated nucleotides from the nascent transcript during rapid chain extension (6). This proofreading ability of Pol I, Pol II, and Pol III can mitigate the possibility of their inhibition by incorporated ribonucleotide analogs. POLRMT, on the other hand, does not have this proofreading activity, which makes it particularly vulnerable to inhibition by incorporated ribonucleotide analog 5′-triphosphates (7).

The crystal structure of POLRMT has already been resolved, and extensive studies have been done to characterize its enzymatic activity (8, 9). POLRMT is a single-subunit DNA-dependent RNA polymerase that is encoded in the nuclear genome. It is evolutionarily related to the bacteriophage T7 class of single-subunit RNA polymerases (RNAPs). It contains a carboxy-terminal polymerase domain that catalyzes the ribonucleotide incorporation reaction and N-terminal structural domains that can interact with other transcriptional factors and regulate transcription. Unlike T7 RNAP, which can initiate transcription alone, POLRMT requires assistance from other accessory factors (mitochondrial transcription factor B2 [TFB2M] and mitochondrial transcription factor A [TFAM]) to accomplish promoter-specific transcription (10). POLRMT is responsible for mitochondrial gene expression (mitochondrial transcription) as well as for the synthesis of RNA primers for the initiation of DNA replication in the mitochondrial genome. Therefore, inhibition of POLRMT leads to the inhibition of both mitochondrial DNA transcription, resulting in decreased mitochondrial protein expression, and mitochondrial DNA replication, resulting in a decrease in the mitochondrial number.

To be substrates for RNA polymerases, ribonucleoside/ribonucleotide analogs must first cross the cell membrane, enter the intracellular compartment, and be converted into their corresponding ribonucleotide analog 5′-triphosphates by cellular kinases. The difference in the cell type-specific or tissue-specific metabolism of ribonucleoside/ribonucleotide analogs can lead to a considerable variation in the observed cell- and tissue-specific toxicity. There is a growing pool of evidence showing that ribonucleotide analog 5′-triphosphates can serve as substrates for human POLRMT and can be incorporated into newly synthesized RNA by POLRMT. Consequently, inhibition of mitochondrial RNA synthesis can lead to the disruption of mitochondrial DNA replication and/or mitochondrial function, causing mitochondrial toxicity (11–16). Therefore, characterization of the incorporation of ribonucleotide analog 5′-triphosphates by POLRMT could provide a useful tool for the evaluation of rNIs to determine their potential mitochondrial toxicity.

In this report, we demonstrate a simplified method to prepare an active recombinant POLRMT in a large quantity and of high quality. Using this purified POLRMT, we developed a robust *in vitro* nonradioactive primer extension assay using a fluorescently labeled RNA primer annealed to a single-stranded DNA template. The incorporation efficiency and the chain termination ability of a series of ribonucleotide analog 5′-triphosphates were tested. The structure-activity information derived from these studies can be used to design rNIs with a lower efficiency of incorporation by POLRMT, thus reducing their potential mitochondrial toxicity.

## RESULTS

### Expression and purification of human POLRMT.

**FIG 1:**
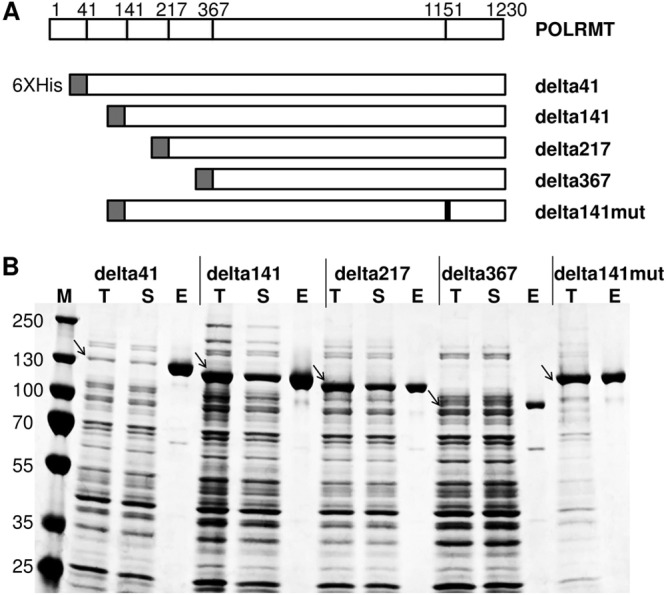
Construction, expression, and purification of different POLRMT variants. (A) Schematic diagram of protein expression vectors. The full length of POLRMT is shown on top. Delta41, delta141, delta217, and delta367 represent expression vectors containing truncations in the N terminus of POLRMTs of different lengths. Delta141mut contains a mutation at amino acid 1151 (D1151A; black vertical line). Dark-gray bar in each construct represents N-terminal 6xHis-tag. (B) SDS-PAGE analysis of protein expression and purification. Lanes T, total cell lysate; lanes S, soluble fraction; lanes E, eluate from the HisPur Ni-NTA agarose resin; lane M, protein molecular mass markers. The sizes of the protein markers (in kilodaltons) are indicated on the left. Arrows indicate expressed proteins.

The human POLRMT gene was amplified from a cDNA clone and cloned into an Escherichia coli expression vector. Different variants of POLRMT (delta41, delta141, delta 217, and delta367) containing N-terminal truncations of various lengths were constructed. Diagrams of the expression vectors are shown in Fig. 1A. A predicted inactive polymerase mutant control, delta141mut, containing an active-site mutation (D1151A) was also prepared. All recombinant proteins contained a 6×His tag at the N terminus to facilitate protein purification. Our results showed that all five POLRMT variants can be successfully expressed in E. coli, and the expression levels and the solubilities of the recombinant proteins varied (Fig. 1B). The expression levels of delta41 and delta367 were lower than the expression levels of delta141 and delta217. Delta41 was largely soluble, while delta141 and delta217 were only partially soluble. The expressed recombinant proteins could easily be purified by nickel affinity purification using HisPur Ni-nitrilotriacetic acid (NTA) agarose resin. After purification, the identity of the purified proteins was determined by mass spectrometry analysis, and the proteins were confirmed to be POLRMT (data not shown).

### Primer extension assay to measure polymerase activity.

**FIG 2:**
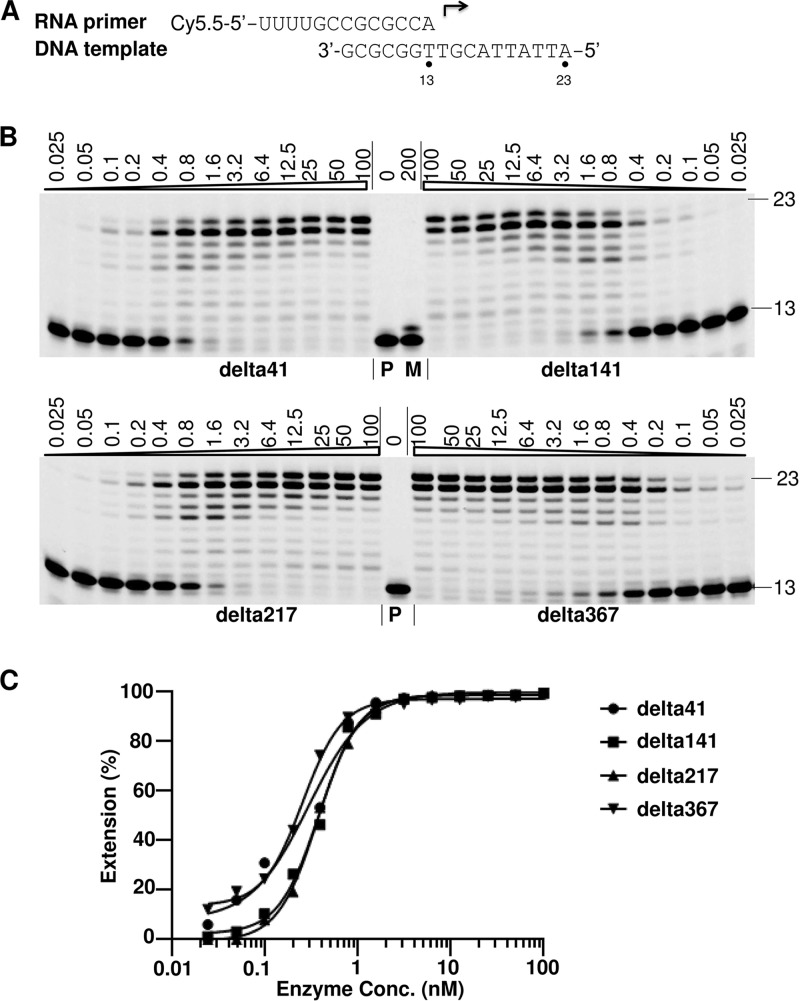
Analysis of POLRMT activity using a primer extension assay. (A) The RNA primer and DNA template used in this assay. The 13-mer primer (top) contains a fluorescent label (Cy5.5) at the 5′ end. The arrow indicates the location and direction of primer extension to form a 23-mer product. (B) Analysis of the polymerase activity of different POLRMT variants (delta41, delta141, delta217, delta367 and delta141mut (M), as indicated at the bottom of each gel). Serial dilutions of POLRMTs (in nanomolar), as indicated on the top of the gel, and a constant concentration of P/T (10 nM) were used in the assay. The reactions were initiated by addition of 100 μM rNTPs; the reactions continued at 22°C for 1 h and were then stopped by adding quenching solution. The products were separated on denaturing polyacrylamide gels. Lanes P, primer control; lane M, reaction with 200 nM delta141mut. The number 13 on the right of the gels indicates the location of the 13-mer unextended primer, and the number 23 indicates the location of the 23-mer full-length product. (C) The percentages of the full-length products in panel B were plotted against the enzyme concentrations. The results were fitted to sigmoidal dose-response curves using the GraphPad Prism program.

To measure the polymerase activity of the purified POLRMTs, a primer extension assay employing a DNA template (17-mer) and a fluorescently labeled RNA primer (13-mer) was developed (Fig. 2A). Briefly, a DNA template and a fluorescently labeled RNA primer-template (P/T) duplex (10 nM) were incubated with different concentrations of POLRMT enzymes in the reaction buffer in the presence of 100 μM rNTPs at 22°C for 1 h. The products of the primer extension were analyzed by denaturing polyacrylamide gel electrophoresis (urea-PAGE). The result showed that the primer was fully extended by delta41, delta141, delta217, and delta367 (Fig. 2B). Only slight RNA synthesis activity was detected using delta141mut (Fig. 2B, lane M), suggesting that the primer extension activity in the reaction mixtures with nonmutated enzymes could be attributed to purified active POLRMTs. To estimate the enzymatic activities of the different variants of POLRMT, the percentage of extended primer in the total reaction mixture was plotted against the log of the enzyme concentrations (Fig. 2C). The results were fit to S-shaped dose-response curves and showed that the curves were similar for different variants of POLRMT, suggesting that the polymerase activities of different POLRMTs were similar. Because the expression of delta141 was better than that of the other constructs and it could easily be purified in a large quantity and was found to be of high quality, the delta141 enzyme was chosen to be tested in the following enzyme characterization and ribonucleotide analog 5′-triphosphate incorporation experiments.

### Effect of P/T scaffold on the primer extension reaction by POLRMT.

**FIG 3:**
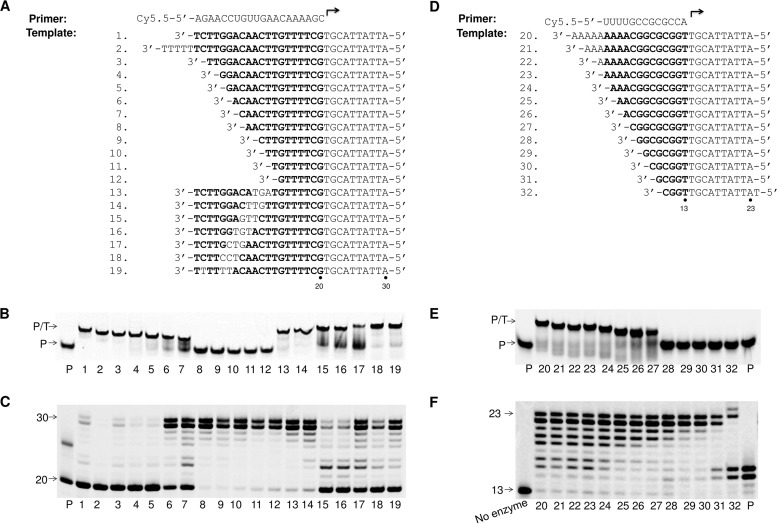
Influence of RNA/DNA scaffolds on the efficiency of the primer extension reaction catalyzed by POLRMT. (A) The RNA primer and DNA templates (templates 1 to 19) used in the experiments whose results are shown in panels B and C. Bold letters in the DNA templates indicate the regions that are complementary with the RNA primer. (B) Native PAGE analysis of the P/T duplexes formed with the RNA primer and the different DNA templates listed in panel A. The number of the DNA template from panel A used for annealing with the RNA primer is indicated under each lane. Lane P, primer alone. The P on the left of the image indicates the migration of the RNA primer, and the P/T indicates the migration of the RNA/DNA duplex. (C) Primer extension assay using the different P/Ts from panel A. Each P/T (10 nM) was used with 20 nM POLRMT. The reactions were initiated by addition of 100 μM rNTPs and continued at 22°C for 1 h, and the products were separated on denaturing polyacrylamide gels. The number of the DNA template from panel A used in each reaction is indicated under each lane. Lane P, primer extension reaction with RNA primer only. The number 20 on the left of the gel indicates the migration of the RNA primer, and the number 30 indicates the migration of the fully extended product. (D) RNA primer and DNA templates (templates 20 to 32) used in the experiments whose results are shown in panels E and F. Bold letters in the DNA templates indicate regions that are complementary with the RNA primer. (E) Native PAGE analysis of the formation of stable P/T duplexes formed with the RNA primer and DNA templates listed in panel D. The number of the DNA template from panel D used for annealing with the RNA primer is indicated under each lane. Lane P, primer alone. The P on the left of the image indicates the migration of the RNA primer, and the P/T indicates the migration of the RNA/DNA duplex. (F) Primer extension assay using the different P/Ts shown in panel D. The reaction conditions were as described in the legend to panel C. The number of the DNA template from panel D used in each reaction is indicated under each lane. No enzyme, primer extension reaction without POLRMT; lanes P, primer extension reaction with RNA primer and no DNA template. The number 13 on the left of the gel indicates the location of RNA primer, and the number 23 indicates the location of the fully extended product.

To measure the effect of the P/T scaffold on the efficiency of the primer extension reaction catalyzed by POLRMT, different DNA templates having different lengths of the sequence that can hybridize with the RNA primer were tested (Fig. 3A). The formation of a stable RNA primer/DNA template (P/T) duplex was analyzed by nondenaturing polyacrylamide gel electrophoresis (Fig. 3B). The stable RNA/DNA duplexes moved slower in the native gel (Fig. 3B, top band labeled P/T) than the primer alone (Fig. 3B, lane P). The results showed that templates 1, 2, 3, 4, 5, 13, 14, 18, and 19 could form stable P/T duplexes, while the P/T duplexes formed with templates 6, 7, 15, 16, and 17 were partially stable. On the other hand, templates 8, 9, 10, 11, and 12 did not form stable P/T duplexes with the RNA primer (Fig. 3B), probably due to a short complementary region. The efficiency of POLRMT-catalyzed primer extension using these P/Ts was tested in a primer extension assay (Fig. 3C). The highest efficiency of primer extension was observed using templates 8, 9, 10, 11, 12, 13, and 14 (Fig. 3C, lanes 8 to 14), the primer extension efficiency was intermediate using templates 6, 7, 17, and 19 (Fig. 3C, lanes 6, 7, 17, and 19), and the primer extension efficiency was very low using templates 1, 2, 3, 4, 5, 15, 16, and 18 (Fig. 3C, lanes 1 to 5, 15, 16, and 18). From these results, it is evident that the length of the P/T double-stranded region is important for the primer extension reaction catalyzed by POLRMT: the optimal length of the double-stranded region was between 7 and 12 nucleotides (Fig. 3C, lanes 8 to 14) for this primer.

To better evaluate the efficiency of primer extension using template 1 and template 9, an enzyme dilution experiment was performed (see Fig. S1A to C in the supplemental material). P/Ts 1 and 9 were extended using different amounts of POLRMT under conditions that were otherwise the same. The result showed that the efficiency of RNA synthesis by POLRMT using template 1 was about ∼16-fold lower than that by POLRMT using template 9.

To test for the effect of the primer/template sequence, a different primer and template set which has been shown to be efficiently utilized by POLRMT in a primer extension assay (17) was also tested in this study (Fig. 3D to F). The results showed that templates 20 to 27 can form stable P/T duplexes with this RNA primer, while templates 28 to 32 cannot (Fig. 3E), and that P/Ts 20 through 30, which form a double-stranded region longer than 6 bp with this primer, can be efficiently utilized by POLRMT in the primer extension reaction (Fig. 3F). These results also confirmed that the efficiency of extension was higher when templates with shorter hybridization regions were used (Fig. 3F, lanes 23 to 30). The efficiency of primer extension using templates 23 and 29 was further evaluated in an enzyme dilution experiment (Fig. S1D to F). The results confirmed that the primer extension efficiency was 3 times higher when shorter template 29 was used compared to the longer template 23.

The results shown in Fig. 3 suggest that the length of the double-stranded region of RNA/DNA duplexes is important for the primer extension performed by POLRMT and that under these *in vitro* conditions POLRMT has a preference for P/T duplexes with short hybridization regions. For the initiation of the primer extension reaction, it is conceivable that the accommodation of shorter and maybe less rigid P/T double-stranded complexes in the POLRMT active site is more efficient than that of longer, more rigid P/T double-stranded complexes.

The 13-mer RNA primer in the last experiment could be extended by POLRMT in the absence of a DNA template (Fig. 3F, lane P). We hypothesized that this RNA primer can form a dimer or a self-priming stem-loop structure and that POLRMT can synthesize RNA using RNA as a template. To test this, the RNA primer alone was incubated with POLRMT in the absence of the DNA template (Fig. S2). The results showed that the primer could be extended only in the presence of ATP, a finding which agrees with the proposed primer extension model (Fig. S2A). This result also suggests that POLRMT can use RNA as a template to synthesize RNA, at least under the *in vitro* conditions used.

### Time course of primer extension reaction with POLRMT.

**FIG 4:**
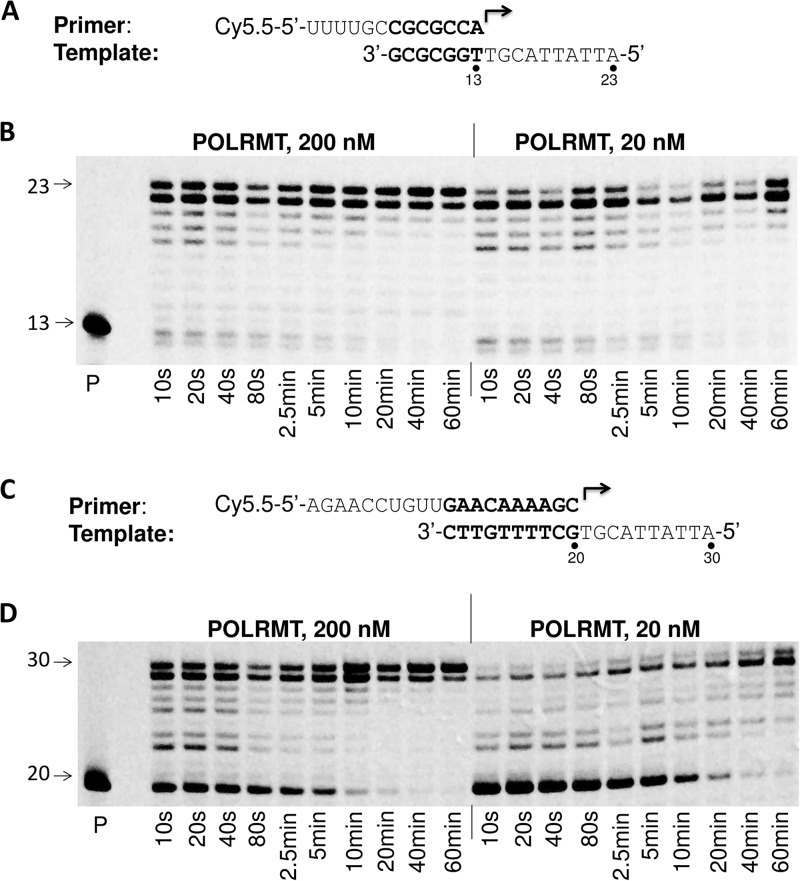
Time courses of POLRMT-catalyzed primer extension reactions with different P/Ts. (A) The rNA primer and DNA template used in the experiment whose results are shown in panel B. (B) Time course of POLRMT-catalyzed primer extension reaction performed using the P/T shown in panel A. Two different concentrations of POLRMT (20 nM and 200 nM; indicated on the top of the gel) and 10 nM P/T were used in the assay. The reactions were initiated by addition of 100 μM rNTP. Five-microliter aliquots were withdrawn at different times after initiation of the reaction, as indicated at the bottom of each lane, and mixed with 5 μl of quenching/loading buffer. The products were analyzed by denaturing PAGE. Lane P, primer extension reaction without NTPs. The number 13 on the left of the gel indicates the migration of the RNA primer, and the number 23 indicates the migration of the fully extended product. (C) The RNA primer and DNA template used in the experiment whose results are shown in panel D. (D) Time course of POLRMT reaction using the P/T shown in panel C. The analysis was done as described in the legend to panel B. The number 20 indicates the migration of the RNA primer, and the number 30 indicates the migration of the fully extended product.

The kinetics of POLRMT-catalyzed primer extension were evaluated by measuring the time course of the reaction (Fig. 4). Two RNA primer and DNA template sets, that were shown in our previous experiments to be efficiently utilized by POLRMT, were used in this assay. When a P/T formed with a 13-mer primer was used, the primer extension reaction was very fast and almost complete within 10 s (Fig. 4A and B). On the contrary, when a P/T formed with a longer (20-mer) primer was used, the initiation of the primer extension reaction was slow (Fig. 4C). A high concentration of the POLRMT enzyme accelerated the reaction to some degree, and the reaction was mostly complete within 10 to 20 min (Fig. 4D). These results suggest that POLRMT prefers P/Ts formed with short RNA primers and/or that the primary structure of the primer may affect the enzyme efficiency.

### Nucleotide analog 5′-triphosphate incorporation and chain termination.

**FIG 5:**
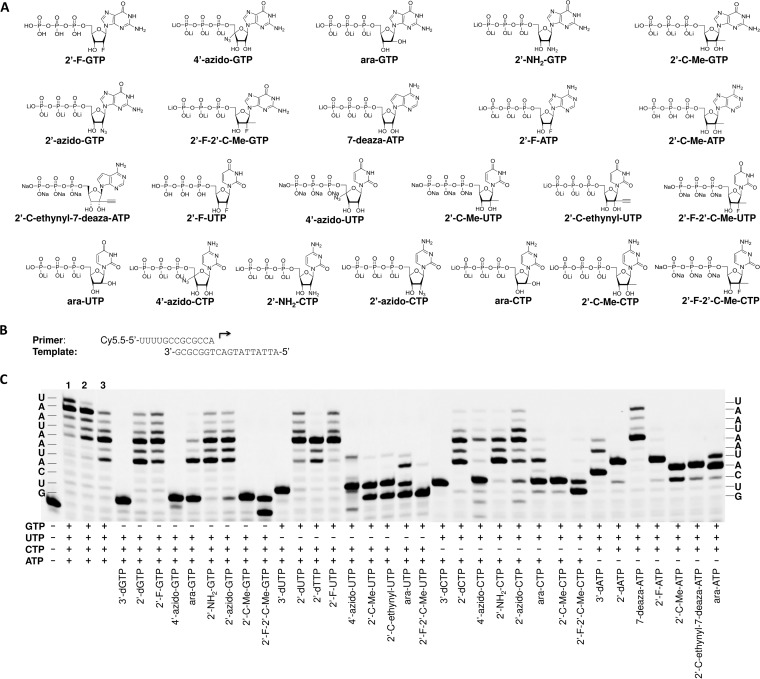
Analysis of chain termination effects of ribonucleotide analogs after incorporation into RNA synthesized by POLRMT. (A) Chemical structures of the ribonucleotide 5′-triphosphate analogs tested in the assay. (B) The primer and template used in the assay whose results are shown here. (C) Analysis of the incorporation and chain termination abilities of the ribonucleotide analogs. Primer extension reactions were initiated by the addition of ribonucleotide analogs at 100 μM and of three complementing natural ribonucleotides at 1 μM each, as indicated under each lane. The reactions were performed at 22°C for 1 h. The products of the primer extension reaction were resolved by denaturing PAGE. The natural ribonucleotides to be incorporated are shown on the sides of the graph. The natural rNTP concentrations in lanes 1, 2, and 3 are 100 μM, 10 μM, and 1 μM, respectively.

The incorporation of ribonucleotide analog 5′-triphosphates into mitochondrial RNA by POLRMT may disrupt mitochondrial RNA and DNA synthesis, which may lead to mitochondrial toxicity. The utilization of different ribonucleotide analog 5′-triphosphates by POLRMT and the ability of the analogs to cause chain termination after incorporation were evaluated in the optimized primer extension assay. Figure 5 shows the incorporation and chain termination potential of several ribonucleotide analog 5′-triphosphates tested in the primer extension assay with POLRMT. In this experiment, primer extensions were performed in the presence of 100 μM of a ribonucleotide analog 5′-triphosphate and 1 μM of three complementing natural rNTPs. The results show that many ribonucleotide analog 5′-triphosphates can be incorporated into synthesized RNA by POLRMT and that the incorporation of some of them can cause chain termination. For GTP analogs, incorporation of 3′-dGTP, 2′-C-Me-GTP, 4′-azido-GTP, and 2′-F-2′-C-Me-GTP caused immediate chain termination; incorporation of ara-GTP caused partial chain termination; incorporation of 2′-azido-GTP caused only weak chain termination; and incorporation of 2′-dGTP and 2′-F-GTP did not lead to chain termination. For UTP analogs, incorporation of 3′-dUTP caused immediate chain termination; incorporation of 4′-azido-UTP and ara-UTP caused partial chain termination; incorporation of 2′-C-Me-UTP and 2′-C-ethynyl-UTP caused strong, but not complete, chain termination; and incorporation of 2′-dUTP and 2′-F-UTP did not cause termination. When 2′-dTTP was tested, the first and second 2′-dTTP could be incorporated without chain termination, but the third 2′-dTTP could not be incorporated. Incorporation of 2′-dUTP and 2′-F-UTP was quite efficient and caused practically no chain termination. No significant incorporation was detected for 2′-F-2′-C-Me-UTP. For CTP analogs, incorporation of 3′-dCTP, 2′-C-Me-CTP, and 2′-F-2′-C-Me-CTP caused immediate chain termination; incorporation of 2′-dCTP and 2′-NH_2_-CTP did not cause immediate chain termination but led to a decrease in the amount of longer RNA products that were formed; incorporation of 4′-azido-CTP or ara-CTP caused partial chain termination; and incorporation of 2′-azido-CTP caused only weak chain termination but led to a decrease in the amount of longer RNA products that were formed. For ATP analogs, 3′-dATP should cause immediate chain termination, and the longer RNA products observed were probably due to the misincorporation of natural rNTPs in place of 3′-dATP. Incorporation of 2′-C-Me-ATP and 2′-C-ethynyl-7-deaza-ATP caused immediate chain termination; incorporation of ara-ATP caused partial chain termination. Incorporation of 2′-dATP or 2′-F-ATP did not lead to immediate chain termination, but the enzyme could not add the second 2′-dATP or 2′-F-ATP to the synthesized RNA. Incorporation of 7-deaza-ATP did not affect RNA synthesis under the assay conditions used. In summary, the incorporation of 2′-C-methyl-, 2′-C-ethynyl-, and 2′-F-2′-C-methyl-modified rNTPs caused efficient chain termination for POLRMT-catalyzed RNA synthesis, and incorporation of 4′-azido-modified rNTPs and ara-NTPs resulted in partial but quite efficient chain termination.

### Incorporation efficiency of ribonucleotide analog 5′-triphosphates by POLRMT.

**FIG 6:**
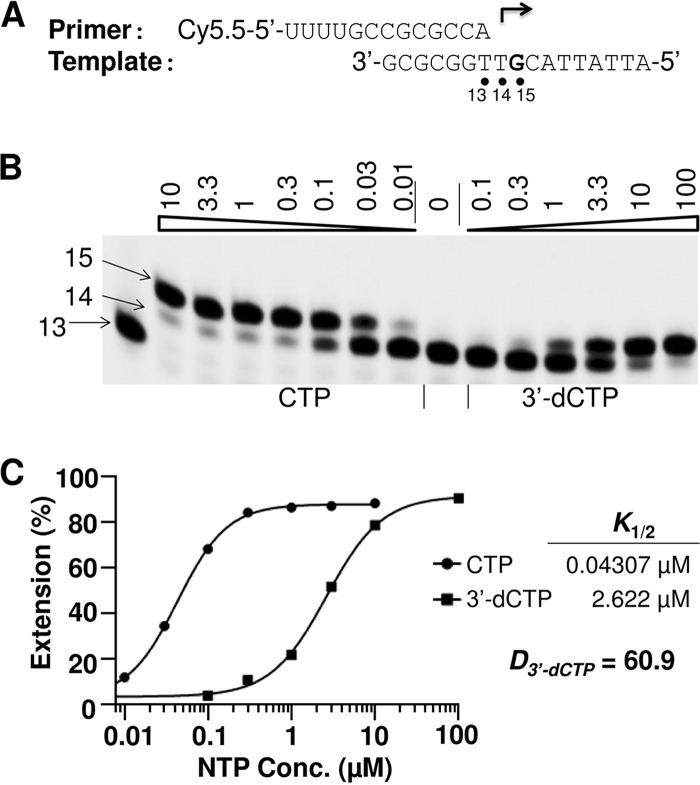
Measurement of the discrimination value of 3′-dCTP. (A) The primer and template used to assay CTP analogs. (B) A representative image of the results of the analysis of *D*_3′-dCTP_ values. POLRMT (20 nM) was incubated with 10 nM P/T and 1 μM ATP (the first ribonucleotide to be incorporated) in reaction buffer for 5 min at 22°C and then rapidly mixed with different concentrations (in micromolar) of 3′-dCTP or CTP, as indicated above each lane. The reactions were continued at 22°C for 30 s before addition of stopping buffer, and the products were resolved by denaturing PAGE. The identity of the tested ribonucleotide is indicated at the bottom of the gel. The locations of the 13-mer primer and 14- and 15-mer first and second ribonucleotide extension products, respectively, are indicated on the left. (C) Quantitative analysis of CTP and 3′-dCTP incorporation in the experiment whose results are shown in panel B. The incorporation efficiency was evaluated on the basis of the extension of 14-mer to 15-mer products. The measured *K_1/2_* values for CTP and 3′-dCTP in this experiment were 0.04307 μM and 2.622 μM, respectively. The discrimination value, *D*_3′-dCTP_, calculated as *K*_1/2, 3′-dCTP_/*K*_1/2, CTP_, is shown on the right of the graph.

Competition with natural rNTPs for incorporation into mitochondrial RNA is believed to be a reason for the mitochondrial toxicity caused by rNIs. Therefore, the incorporation efficiency of ribonucleotide analog 5′-triphosphates by POLRMT may serve as an important criterion to evaluate ribonucleoside/ribonucleotide analogs for potential mitochondrial toxicity. The discrimination (*D*) value, defined as the efficiency of incorporation of a natural rNTP over the efficiency of incorporation of a ribonucleotide analog 5′-triphosphate, has been used to compare the incorporation efficiency of different ribonucleotide analog 5′-triphosphates by Zika virus and dengue virus RNA-dependent RNA polymerases (RdRps) (18). Using a similar method, the discrimination values of ribonucleotide analog 5′-triphosphates, measured in the established POLRMT primer extension assays, were employed to evaluate the relative incorporation efficiency of ribonucleotide analog 5′-triphosphates by POLRMT. In the initial experiments, the incorporation efficiency of 3′-deoxynucleoside triphosphate (3′-dNTP) by POLRMT was measured, and the corresponding discrimination values for 3′-dNTP (*D*_3′-dNTP_) were calculated. The time course experiment described above (Fig. 4) showed that the incorporation of natural rNTPs by POLRMT is fast when nucleotides are used at 100 μM, and in order to get a quantitative measurement of the concentration resulting in 50% product extension (*K*_1/2_) for natural rNTP, a short reaction time (15 to 60 s) was used. Examples of the primer and template design and of the results of testing of CTP and 3′-dCTP are shown in Fig. 6. In this assay, the measured values of *K*_1/2_ (the nucleotide concentration at which half of the 14-mer product is extended to the 15-mer product) for CTP (*K*_1/2, CTP_) and 3′-dCTP (*K*_1/2, 3′-dCTP_) were 0.04307 μM and 2.622 μM, respectively, and the calculated discrimination value (*D*_3′-dCTP_ = *K*_1/2, 3′-dCTP_/*K*_1/2, CTP_) was 60.9 (Fig. 6C). The discrimination values for 3′-dATP, 3′-dGTP, and 3′-dUTP were measured using similar methods (Fig. S3), and the results are summarized in Table 1. In the tests with *D*_3′-dUTP_ and *D*_3′-dGTP_, two different reaction times (30 s and 60 s for *D*_3′-dUTP_ and 30 s and 15 s for *D*_3′-dGTP_) were tested and resulted in similar discrimination values, which suggests that the discrimination values measured using this method are not dependent on the reaction time used in the assay.

**TABLE 1:** Discrimination values for 3′-dCTP, 3′-dATP, 3′-dUTP, and 3′-dGTP^*a*^

Nucleotide analog	Repeat I			Repeat II			*D*_3′d-NTP_ (mean ± SD)
	Time (s)	*K*_1/2_ (μM)	*D*_3′-dNTP_	Time (s)	*K*_1/2_ (μM)	*D*_3′-dNTP_	
ATP	30	0.09286		30	0.09298		
3′-dATP	30	1.926	20.7	30	1.977	21.3	21.0 ± 0.4
CTP	30	0.04503		30	0.04307		
3′-dCTP	30	2.637	58.6	30	2.622	60.9	59.7 ± 1.6
GTP	30	0.01373		15	0.02737		
3′-dGTP	30	0.4655	33.9	15	0.9601	35.1	34.5 ± 0.8
UTP	30	0.2226		60	0.106		
3′-dUTP	30	22.34	100.4	60	9.439	89.0	94.7 ± 8.0

a *D*_3′-dNTP_ is equal to *K*_1/2, *3*′-dNTP_/*K*_1/2, rNTP_. Time indicates the primer extension reaction time in each experiment.

**FIG 7:**
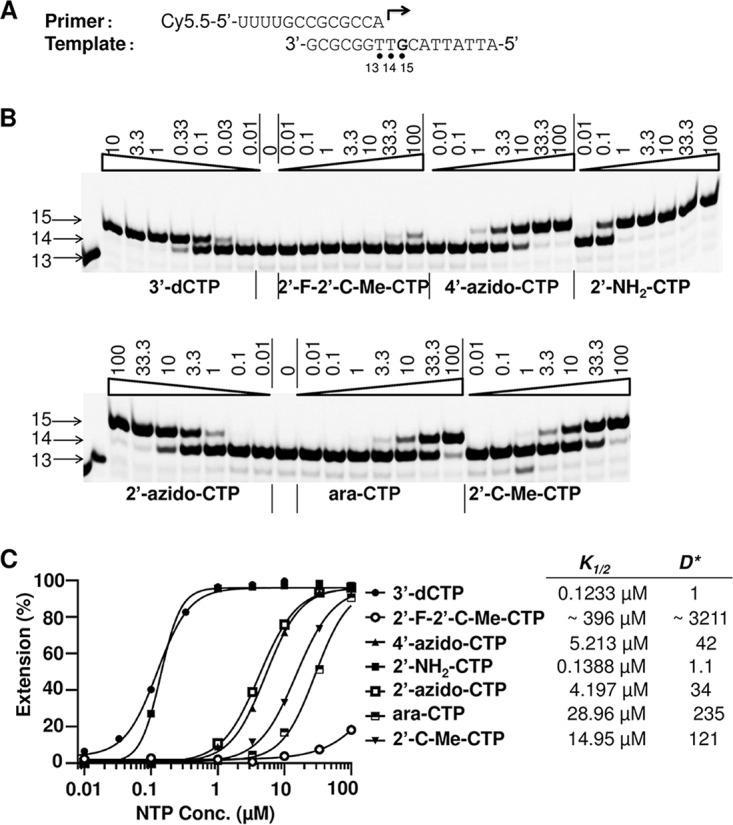
Measurement of the discrimination values of the CTP analogs. (A) The primer and template used to assay the CTP analogs. (B) A representative image of the results of the analysis of the CTP analogs. Primer extension reaction mixtures contained 10 nM P/T and 20 nM POLRMT, and the reactions were performed in the presence of 1 μM ATP as the first ribonucleotide and increasing concentrations (in micromolar) of 3′-dCTP or CTP analogs, as indicated above each lane. The reactions were performed at 22°C for 30 min, and the products were resolved by denaturing PAGE. The identity of the tested ribonucleotide is indicated at the bottom of the gel. The migrations of the 13-mer primer and the 14- and 15-mer first and second ribonucleotide extension products, respectively, are indicated on the left. (C) Quantitative analysis of CTP analogs and 3′-dCTP incorporation. The incorporation efficiency was evaluated on the basis of the extension of 14-mer to 15-mer products. The measured *K_1/2_* values are shown on the right of the graph. The discrimination between CTP analogs and 3′-dCTP ${D*}_{\text{analog}}$ was calculated as *K*_1/2, analog_/*K*_1/2, 3′dCTP_, and the values are shown on the right of the graph. The discrimination between CTP analogs and natural CTP ${D^{cal}}_{\text{analog}}$ was calculated as ${D*}_{\text{analog}}\times D_{3\prime \text{-dCTP}}$. *D*_3′-dCTP_ is 59.7 ± 1.6 (Table 1), so for 2′-F-2′-C-Me-CTP, 4′-azido-CTP, 2′-NH_2_-CTP, 2′-azido-CTP, ara-CTP, and 2′-C-Me-CTP, the calculated values of ${D^{cal}}_{\text{analog}}$ in this experiment were 191,696, 2,507, 66, 2030, 14,029, and 7,224, respectively.

The incorporation efficiency of ribonucleotide analog 5′-triphosphates by POLRMT was much lower than that of natural rNTPs. A longer reaction time (30 to 60 min) was needed to increase the percentage of incorporation of the ribonucleotide analog 5′-triphosphates by POLRMT and to get a quantitative measurement of their *K*_1/2_ values. Since the *K*_1/2_ of natural CTP is impossible to measure directly under such conditions (it is below the limit of sensitivity of the assay), the *K*_1/2_ value of 3′-dCTP was used as a surrogate comparator. An example of testing of CTP analog incorporation by POLRMT is shown in Fig. 7. In this assay, the *K*_1/2_ values of several CTP analogs were measured and compared to the *K*_1/2_ value of 3′-dCTP, which was used as a reference to calculate the ${D*}_{\text{CTP analog}}$ value (where ${D*}_{\text{CTP analog}\;}=K_{1/2,\;\text{CTP analog}}/K_{1/2,\;3\prime \text{-sCTP}}$). The discrimination values of the different ribonucleotide analog 5′-triphosphates tested in this study are summarized in Table 2. *D*^cal^ is a calculated discrimination value obtained using the equation ${D^{cal}}_{\text{NTP analog}\;}={D*}_{\text{NTP analog}\;}\times D_{3\prime \text{-dNTP}}$, where the *D*_3′-dNTP_ values are from Table 1. *D*^cal^ represents the discrimination of incorporation by POLRMT between natural rNTP and a tested ribonucleotide analog 5′-triphosphate. The misincorporation frequency of ribonucleotide analog 5′-triphosphates was also calculated as $1/{D^{cal}}_{\text{analog}}$, which represents the incorporation frequency of ribonucleotide analog 5′-triphosphates when equal concentrations of the tested ribonucleotide analog 5′-triphosphate and the corresponding natural rNTP are used. Our results show that many ribonucleotide analog 5′-triphosphates can be incorporated by POLRMT, and the incorporation efficiency varies widely between the analogs. The structure-activity information derived from comparison of the *D*^cal^ values of different ribonucleotide analog 5′-triphosphates can provide a guideline for designing ribonucleoside/ribonucleotide analogs with lower efficiencies of incorporation by POLRMT. For example, 2′-F-modified ribonucleotide analogs have ∼1,000- to 5,000-fold lower incorporation efficiencies than their natural counterparts, which suggests that 2′-F-modified ribonucleotide analogs may have less mitochondrial toxicity.

**TABLE 2:** Discrimination values for ribonucleotide analogs by POLRMT^*a*^

Nucleotide analog	${D*}_{\text{analog}}$ (mean ± SD)	${D^{\text{cal}}}_{\text{analog}}$ (mean ± SD)	Misincorporation frequency
ATP analogs			
3′-dATP	1	21 ± 0.4	(4.8 ± 0.09) × 10^−2^
2′-dATP	375 ± 57	7,875 ± 1,197	(1.3 ± 0.2) × 10^−4^
2′-F-ATP	240 ± 17	5,040 ± 357	(2.0 ± 0.1) × 10^−4^
7-deaza-ATP		2.95 ± 0.07	(3.4 ± 0.08) × 10^−1^
2′-C-Me-ATP	123 ± 8	2,583 ± 168	(3.9 ± 0.3) × 10^−4^
2′-C-ethynyl-7-deaza-ATP	107 ± 4	2,247 ± 84	(4.5 ± 0.2) × 10^−4^
ara-ATP	226 ± 16	4,746 ± 336	(2.1 ± 0.1) × 10^−4^
GTP analogs			
3′-dGTP	1	34.5 ± 0.8	(2.9 ± 0.07) × 10^−2^
2′-dGTP	82.2 ± 8.3	2,836 ± 286	(3.5 ± 0.4) × 10^−4^
2′-F-GTP	44.7 ± 4.7	1,542 ± 162	(6.5 ± 0.7) × 10^−4^
2′-C-Me-GTP	83.0 ± 8.9	2,864 ± 307	(3.5 ± 0.4) × 10^−4^
2′-F-2′-C-Me-GTP	∼7,618 ± 3,042	∼262,821 ± 104,949	(∼3.8 ± 1.5) × 10^−6^
2′-NH_2_-GTP	2.0 ± 0.4	69 ± 13.8	(1.4 ± 0.3) × 10^−2^
2′-azido-GTP	115 ± 13	3,968 ± 449	(2.5 ± 0.3) × 10^−4^
ara-GTP	18.8 ± 0.1	649 ± 3	(1.5 ± 0.007) × 10^−3^
4′-azido-GTP	0.6 ± 0.1	20.7 ± 3.45	(4.8 ± 0.8) × 10^−2^
CTP analogs			
3′-dCTP	1	59.7 ± 1.6	(1.7 ± 0.04) × 10^−2^
2′-dCTP	77.2 ± 3.5	4,609 ± 209	(2.2 ± 0.1) × 10^−4^
2′-F-CTP	22.7 ± 0.2	1,355 ± 12	(7.4 ± 0.07) × 10^−4^
2′-NH_2_-CTP	1.2 ± 0.1	71.6 ± 6	(1.4 ± 0.1) × 10^−2^
2′-azido-CTP	32.8 ± 1.8	1,958 ± 107	(5.1 ± 0.3) × 10^−4^
Ara-CTP	241 ± 8	14,388 ± 478	(7.0 ± 0.2) × 10^−5^
2′-F-2′-C-Me-CTP	∼3,569 ± 504	∼213,069 ± 30,089	(∼4.7 ± 0.7) × 10^−6^
2′-C-Me-CTP	116 ± 7	6,925 ± 418	(1.4 ± 0.09) × 10^−4^
4′-azido-CTP	44.9 ± 4.1	2,681 ± 245	(3.7 ± 0.3) × 10^−4^
UTP analogs			
3′-dUTP	1	94.7 ± 8	(1.1 ± 0.09) × 10^−2^
2′-dUTP	10.6 ± 4.7	1,004 ± 445	(1.0 ± 0.4) × 10^−3^
2′-dTTP	35.2 ± 7.8	3,333 ± 739	(3.0 ± 0.7) × 10^−4^
2′-F-UTP	7.4 ± 1.6	701 ± 152	(1.4 ± 0.3) × 10^−3^
2′-F-2′-C-Me-UTP	No incorporation	NA	NA
2′-C-Me-UTP	714 ± 80	67,616 ± 7,576	(1.5 ± 0.2) × 10^−5^
2′-C-ethynyl-UTP	697 ± 50	66,006 ± 4,735	(1.5 ± 0.1) × 10^−5^
ara-UTP	∼1,173 ± 135	∼111,083 ± 12,785	(∼9.0 ± 1) × 10^−6^
4′-azido-UTP	3.5 ± 0.8	331 ± 76	(3.0 ± 0.7) × 10^−3^

a ${D*}_{\text{analog}}=K_{1/2,\;\text{analog NTP}}/K_{1/2,\;3\prime \text{-}\text{dNTP}};\;{D^{cal}}_{\text{analog}}={D*}_{\text{analog}}\times D_{3\prime \text{-dNTP}}$, where the values of *D*_3′-dNTP_ are from Table 1. Misincorporation efficiency is equal to $1/{D^{cal}}_{\text{analog}}$. The data are from 2 or 3 independent experiments.

### Fidelity of POLRMT (misincorporation of natural rNTPs).

**FIG 8:**
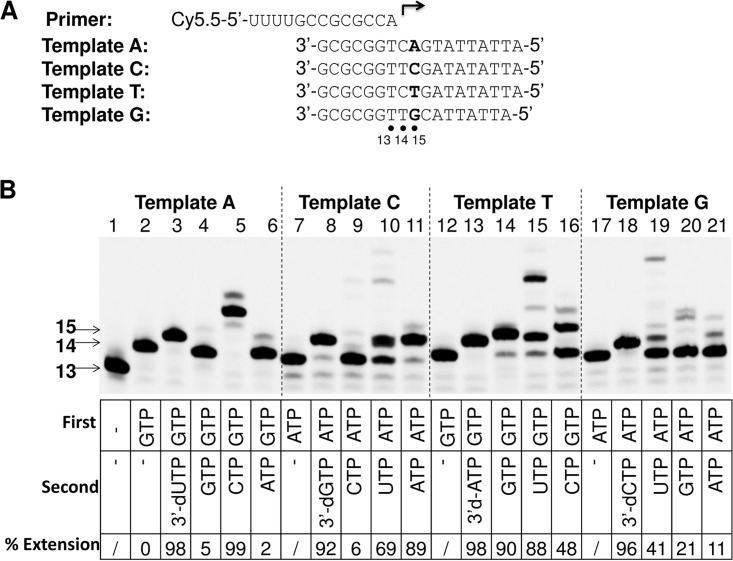
Misincorporation of natural ribonucleotides by POLRMT. (A) The primer and templates used in the assay. (B) An image of the results of primer extension assays performed with the highest NTP concentrations tested that demonstrated the misincorporation of natural rNTP under conditions of different mispairs. The templates used in the reactions are indicated on the top of the gel. The natural rNTPs and 3′-dNTP used in each reaction are indicated at the bottom of each lane. “First” indicates the first correct nucleotide incorporated; “second” indicates the second nucleotide incorporated (misincorporated). POLRMT (20 nM) and 10 nM P/T were used in the assay. The concentration of the first ribonucleotide to be incorporated was 1 μM, and the concentrations of the "second" 3′-dNTPs or natural rNTPs to be incorporated were 100 μM and 1,000 μM, respectively. The reactions were performed at 22°C for 1 h, and the products were resolved by denaturing PAGE. The locations of the 13-mer primer and of the 14- and 15-mer first and second nucleotide extension products, respectively, are indicated on the left. The efficiency of extension was evaluated on the basis of the extension of the 14-mer products as a percentage of the disappearance of the 14-mer in the reactions and is indicated as percent extension.

During transcription, POLRMT has to discriminate between natural rNTPs that are complementary to the template and those that are not complementary to the template in order to prevent transcription errors. The discrimination between natural rNTPs can be used as a guide to evaluate a safe, or natural, level of misincorporation during RNA synthesis by POLRMT, particularly when nucleotide analog 5′-triphosphates cause no, or weak, chain termination. In this experiment, 12 possible mispairs of natural ribonucleotides were tested in a primer extension assay. Four different templates were used to assay the different mispairs (Fig. 8A). In each case, the first ribonucleotide to be incorporated was the correct ribonucleotide and the second ribonucleotide to be incorporated contained different mispairs. For example, lane 2 in Fig. 8B shows extension products with GTP only (the first nucleotide); lanes 3 to 6 show the ability of different NTPs to base pair with deoxyadenosine in template A: 3′-dUTP (a positive control), GTP, CTP and ATP, respectively. The extension of 14-mer RNA synthesized on template A with 3′-dUTP and CTP was efficient (∼98 and 99%, respectively; Fig. 8B, lanes 3 and 5), but it was not efficient when GTP and ATP were paired with deoxyadenosine residue (∼5 and 2%, respectively; Fig. 8B, lanes 4 and 6). The high efficiency of extension with CTP (99%) suggests that CTP can efficiently mispair with deoxyadenosine in the template (CTP-dA). The efficiency of CTP mispairing with deoxyadenosine in the template was further evaluated by measuring the discrimination value for CTP under the condition of base paring with deoxyadenosine using the method described in the previous section. The discrimination of CTP versus UTP when paired with dA was measured to be (1.31 ± 0.17) × 10^4^-fold, which suggests that the misincorporation frequency of CTP base paired with deoxyadenosine in the template was (7.63 ± 0.99) × 10^−5^. The discrimination and misincorporation frequency values of all natural rNTPs under the conditions of different mispairs are summarized in Table 3.

**TABLE 3:** Discrimination values of natural NTPs in different mispairs^*a*^

NTP-template^*b*^	*D* value	Misincorporation frequency^*c*^
GTP-A	—^*d*^	—
CTP-A	(1.31 ± 0.17) × 10^4^	(7.63 ± 0.99) × 10^−5^
ATP-A	—	—
CTP-C	—	—
UTP-C	(8.37 ± 0.056) × 10^5^	(1.19 ± 0.01) × 10^−6^
ATP-C	(4.29 ± 0.60) × 10^5^	(2.33 ± 0.33) × 10^−6^
GTP-T	(6.19 ± 1.30) × 10^4^	(1.62 ± 0.34) × 10^−5^
UTP-T	(4.24 ± 0.68) × 10^4^	(2.36 ± 0.38) × 10^−5^
CTP-T	(3.96 ± 1.20) × 10^5^	(2.53 ± 0.77) × 10^−6^
UTP-G	(1.23 ± 0.051) × 10^6^	(8.13 ± 0.34) × 10^−7^
GTP-G	—	—
ATP-G	—	—

a The discrimination (*D*) values of rNTPs having mispairing extension percentages of 30% or above (as shown in Fig. 8B) were measured using the method described in the legends to Fig. 6 and 7.

b The tested mispairing is indicated as the rNTP-deoxynucleotide pair.

c The misincorporation frequency is equal to 1/*D*. Data are from 2 independent experiments and are reported as the mean ± SD.

d —, the misincorporation efficiency is very low and the *K*_1/2_ values could not be measured quantitatively, so the corresponding *D* values are not provided here.

## DISCUSSION

Different methods have been tried in order to express and purify the active form of recombinant human POLRMT (17, 19, 20), and many of those methods are complicated and require special expression and purification systems. This makes the application of POLRMT for routine screening of new compounds to identify potential mitochondrial toxicity prohibitively expensive. In this report, we showed that an active POLRMT can be expressed in E. coli and purified in a large quantity and to a high quality using a very simple method. Initially in our study, the full-length POLRMT containing the N-terminal mitochondrial localization domain (amino acids [aa] 1 to 42) was constructed, but the full-length recombinant POLRMT could not be expressed in E. coli (data not shown). The mitochondrial localization signal may decrease the stability of POLRMT when it is expressed *in E. coli*. Study of the POLRMT crystal structure showed that, similar to T7 RNA polymerase, POLRMT contains a catalytic carboxy-terminal domain (aa 647 to 1230), an N-terminal domain (aa 368 to 647), a pentatricopeptide repeat domain (aa 218 to 368), and an N-terminal extension (aa 42 to 218) (8). The polymerase domain of POLRMT is located at the C terminus, and the N-terminal domains of POLRMT play a role in interacting with other factors, such as TFB2M and TFAM, which are required for the process of cellular RNA transcription from double-stranded DNA. Since the POLRMT enzyme prepared in our study was intended to be used in an *in vitro* primer extension assay using a short RNA primer annealed to a single-stranded DNA template, the N terminus of POLRMT was deemed to be not essential for the primer extension activity. In this study, a set of N-terminal truncation variants of POLRMT was constructed and tested. The truncation of 141 or 217 amino acids from the N terminus greatly increased the level of recombinant POLRMT expression in E. coli. These truncated enzymes were also capable of performing the primer extension reaction. Different truncated POLRMTs showed similar primer extension activities (Fig. 2). The substrate specificity of three truncated POLRMTs (delta41, delta141, and delta217) was also compared by measuring the discrimination values for 3′-dCTP, 2′-dCTP, and 2′-C-Me-CTP, and for the three enzymes the measured discrimination values for each nucleotide analog were similar (data not shown). Based on these results, we chose the delta141 enzyme for all further tests. It is recognized that, in the cells, the mitochondrial transcription machinery is a multicomponent system consisting of the mitochondrial RNA polymerase, TFB2M, TFAM, and other cellular factors (10, 21). Whether TFB2M, TFAM, and/or other factors have any influence on the substrate specificity of POLRMT was not addressed in this study and needs further investigation.

The results of a few biochemical studies have been published which characterized the enzymatic properties of POLRMT, including the interaction of POLRMT with RNA/DNA scaffolds. Studies done by Smidansky et al. revealed that POLRMT prefers to initiate the primer extension reaction using a 9-bp duplex scaffold and that the length of the RNA/DNA duplex is a major determinant of the stability of POLRMT-nucleic acid complexes (17). T7 RNA polymerase, which is evolutionarily related to POLRMT, has been shown to bind very efficiently an RNA/DNA scaffold with a hybrid region as short as 8 bp, and the length of the hybrid region is essential for this binding (22). In our study, we also showed that the length of the hybridization region in the RNA/DNA duplex is essential for the efficiency of the primer extension reaction by POLRMT and that POLRMT prefers RNA/DNA duplexes having even shorter hybridization regions. The formation of a hybridization region between the RNA primer and the DNA template of only 6 bp was enough for POLRMT to initiate an efficient primer extension reaction, and the efficiency obtained using this short scaffold was higher than that obtained using scaffolds with longer hybridization regions (Fig. 3F, lanes 23 to 30). Interestingly, we observed that it is not necessary to form stable RNA/DNA duplexes in order to initiate a primer extension reaction with POLRMT (Fig.3F, lanes 28-30). Even in cases in which a stable P/T complex can be formed, the preannealing of the RNA primer and DNA template was not necessary (data not shown). It is conceivable that POLRMT can stabilize and/or promote the formation of an RNA/DNA complex during the initiation process or that POLRMT can initially bind single-stranded DNA first (which is present in the reaction mixture in excess) and thus promote annealing of an RNA primer to the DNA template to form a stable RNA/DNA-POLRMT complex. Further experiments need to be carried out to characterize the process of assembly of the transcription initiation complex by POLRMT.

The analog incorporation assay showed that many ribonucleotide analog 5′-triphosphates can be incorporated into synthesized RNA by POLRMT. Many 2′-methyl- and 2′-ethynyl-modified ribonucleotide analog 5′-triphosphates, which are often competitive alternative substrates for viral RNA-dependent RNA polymerases and usually cause chain termination, can also be incorporated by POLRMT and cause chain termination. This result suggests that these ribonucleotide analog 5′-triphosphates have the potential to inhibit mitochondrial RNA synthesis, which may lead to mitochondrial toxicity.

Interestingly, with a few ribonucleotide analog 5′-triphosphates, we observed that the incorporation of a wrong ribonucleotide into RNA may affect not only the incorporation of the next ribonucleotide but also the incorporation of the next several ribonucleotides. For instance, incorporation of 2′-dATP (against dT) did not alter the incorporation efficiency of the following ribonucleotide (UTP-dA), but POLRMT could not subsequently incorporate another 2′-dATP against dT (Fig. 5C). This suggests that the incorporation of a wrong nucleotide may cause a distortion of either the RNA/DNA structure or the POLRMT conformation, which leads to a change in the enzyme's substrate specificity (to 2′-dATP, in this case). It is conceivable that POLRMT has a mechanism to detect minor changes in the upstream RNA during synthesis: when POLRMT detects such changes in the synthesized RNA, it changes/increases the substrate specificity requirements, which, in turn, prevent it from making mistakes repeatedly and, thus, protects the RNA transcripts from the accumulation of misincorporations.

The incorporation efficiency and discrimination values for ribonucleotide analog 5′-triphosphates are usually evaluated in pre-steady-state kinetic studies to measure kinetic parameters (the apparent dissociation constant [*K_d_*_, app_] and the polymerization constant [*K*_pol_]) in the polymerization reaction (17, 23). These studies usually require specialized equipment and take a significant amount of time to perform. In this study, we provide a simple and robust gel-based method to evaluate the incorporation efficiency of ribonucleotide analog 5′-triphosphates by POLRMT. Using this method, we were able to quickly screen a large number of ribonucleotide analog 5′-triphosphates. During the development of this assay, we performed pre-steady-state kinetic studies on the incorporation of several CTP analogs (3′-dCTP, 4′-azido-CTP, 2′-C-Me-CTP, 2′-dCTP, and ara-CTP) by POLRMT to measure their kinetic parameters (*K_d_*_, app_, *K*_pol_) and to calculate their catalytic efficiencies (*K*_pol_/*K_d_*_, app_) (see Fig. S4 in the supplemental material). Because the incorporation of CTP by POLRMT is very fast, the corresponding *K_d_*_, app_ and *K*_pol_ values could not be measured using our standard assay. Using 3′-dCTP as a reference, the discrimination (*D*) values for several CTP analogs were calculated on the basis of the measured catalytic efficiencies and were compared with the D* values in Table 2 measured using our simplified method (Fig. S4H). The comparison showed that for each of the CTP analogs, the calculated *D* values were not exactly the same. One explanation is that the highest CTP analog concentration used in the assays was close to or much lower than its *K_d_*_, app_ values and the *K_d_*_, app_ and *K*_pol_ values were generated using an incomplete curve. That leads to extrapolated and, therefore, not very accurate data. Despite this discrepancy and limitation, the overall trend of the *D* values measured using the two different methods was similar, suggesting that the discrimination value of ribonucleotide analog 5′-triphosphates measured using our simplified method can well represent the incorporation efficiencies of ribonucleotide analog 5′-triphosphates by POLRMT.

The incorporation efficiencies of ribonucleotide analog 5′-triphosphates by POLRMT were usually much lower than those in a similar study on the incorporation of ribonucleotide analog 5′-triphosphates by Zika virus RNA-dependent RNA polymerase (18). For example, the discrimination value of 2′-C-ethynyl-UTP measured in POLRMT assay was ∼66,000, which suggests that the misincorporation frequency of 2′-C-ethynyl-UTP into mitochondrial RNA is about 1.5 × 10^−5^ when the UTP and 2′-C-ethynyl-UTP concentrations are equal. It has been shown that a phosphoramidate prodrug of 2′-C-ethynyl-uridine may lead to mitochondrial toxicity in dogs after 8 days of treatment (12), which suggests that ribonucleotide analog 5′-triphosphates with a discrimination value as high as 66,000 may not be considered safe drugs for mitochondria. In order to use the POLRMT discrimination value as a criterion to evaluate/predict the potential mitochondrial toxicity of tested ribonucleotide analogs, it would be beneficial to have a set of reference ribonucleotide analogs which do and which do not cause mitochondrial toxicity. Currently, it is well accepted that sofosbuvir does not cause mitochondrial toxicity. For sofosbuvir 5′-triphosphate (2′-F-2′-C-methyl-UTP) tested at concentrations up to 500 μM, there was no detectable incorporation into RNA by POLRMT during a 1-h reaction, and if the reaction time was extended to 24 h, only slight incorporation (∼5%) was observed (data not shown). It is practically impossible to generate a discrimination value for sofosbuvir triphosphate using the assay described here. Ribavirin 5′-triphosphate, another ribonucleotide analog 5′-triphosphate that is considered to not cause mitochondrial toxicity, was also tested in the POLRMT primer extension assay. No incorporation was detected during the 1-h reaction with ribavirin 5′-triphosphate concentrations of up to 500 μM (data not shown). On the other hand, transcription errors may occur (and probably do occur) when a wrong natural ribonucleotide is misincorporated into RNA by POLRMT. Such errors may lead to the generation of nonfunctional RNA transcripts. We measured the discrimination values of natural misincorporated ribonucleotides to use those numbers as a guide for prediction of the potential safe levels of misincorporation for new ribonucleotide analogs. A ribonucleotide analog with a discrimination value higher than that of a natural ribonucleotide during mispairing would be considered a safe compound. Our data showed, for example, that CTP mispairing with deoxyadenosine in the template has the highest misincorporation frequency, (7.69 ± 0.97) × 10^−5^ [the discrimination value for this wrong CTP was (1.31 ± 0.17) × 10^4^; Table 3]. Based on these data and references from other studies (12, 13, 16), we have proposed the following criteria for evaluating the potential mitochondrial toxicity of ribonucleotide analogs: a safe compound has a *D* value of >10^5^, a potentially toxic compound has a *D* value of >10^4^ but <10^5^, and a toxic compound has a *D* value of <10^4^, where the *D* value is the value of discrimination of a ribonucleotide analog 5′-triphosphate versus a natural rNTP measured in the POLRMT assay. These criteria are applicable to ribonucleotide analog 5′-triphosphates that do not cause chain termination; these threshold values would be expected to be lower for ribonucleotide analog 5′-triphosphates causing chain termination.

### Conclusion.

This report provides a simple method of POLRMT enzyme preparation and a simple screening method to measure the incorporation efficiency of ribonucleotide analog 5′-triphosphates by POLRMT, which can be used to evaluate the potential mitochondrial toxicity of the corresponding ribonucleoside/ribonucleotide analogs. The methods described here should be very valuable to evaluate candidate rNIs in early stages of discovery and development before a commitment to extensive preclinical and clinical development of the compounds is made.

After submission of this paper for publication, Sultana et al. published a paper (24) presenting measurements of natural nucleotide selectivity/misincorporation by POLRMT obtained using traditional biochemical methods. Similar to the data presented in this paper, Sultana at al. also observed general high discrimination when natural purine nucleotides were incorporated against purines in the template. One significant discrepancy between our results and those in the paper of Sultana et al. was the efficient incorporation of GTP against dA, which was more efficient than that in the CTP-dA pair. Our data suggest that the rate of GTP misincorporation against dA is much lower than that of the CTP-dA pair. A few parameters may have affected the results, such as the use of a double-stranded DNA template rather than a single-stranded DNA template, the primer/template composition, or the measurement of first nucleotide incorporation (the initiation process) rather than second nucleotide incorporation (the elongation), to name a few. Future mechanistic experiments may address this issue.

## MATERIALS AND METHODS

### Chemicals and consumables.

ATP, UTP, CTP, GTP, 3′-dUTP, 3′-dATP, 3′-dGTP, 3′-dCTP, 2′-dATP, 2′-dCTP, 2′-dGTP, 2′-dTTP, 2′-F-CTP, 2′-F-GTP, 2′-NH_2_-GTP, 2′-NH_2_-CTP, 2′-F-ATP, 2′-F-UTP, ara-GTP, ara-ATP, 7-deaza-ATP, ara-CTP, ara-UTP, 2′-azido-GTP, and 2′-azido-CTP were purchased as 100 mM solutions from TriLink Biotechnologies (San Diego, CA). 2′-C-ethynyl-7-deaza-ATP was purchased from Carbosynth (San Diego, CA). Urea, taurine, dithiothreitol (DTT), imidazole, MgCl_2_, and IPTG (isopropyl-β-d-thiogalactopyranoside) were purchased from Sigma (St. Louis, MO). LB medium, bovine serum albumin (BSA), NaCl, Triton X-100, glycerol, protease inhibitor cocktail, Tris-HCl (pH 7.5), and HisPur Ni-NTA agarose resin were purchased from Thermo Fisher Scientific (Waltham, MA). An In-Fusion HD cloning kit and Stellar competent cells were purchased from Clontech (Mountain View, CA). The pMal-c5X vector was purchased from New England Bioscience (NEB; Ipswich, MA). Fluorescently labeled RNA (Cy5.5-RNA) and unlabeled DNA oligonucleotides were chemically synthesized and purified by high-performance liquid chromatography by Integrated DNA Technologies (Coralville, IA). 2′-C-Me-ATP, 2′-C-Me-GTP, 2′-F-2′-C-Me-GTP, 4′-azido-GTP, 2′-F-2′-C-Me-CTP, 2′-C-Me-CTP, 4′-azido-CTP, 2′-F-2′-C-Me-UTP, 2′-C-Me-UTP, 2′-C-ethynyl-UTP, and 4′-azido-UTP were custom synthesized in-house.

### Plasmid construction and protein expression.

Different truncation variants of human POLRMT were PCR amplified from a plasmid carrying POLRMT cDNA (GenBank accession no. BC098387; clone identifier, 5264127; Dharmacon, CO) and cloned into the pMal-c5X vector under the control of the *tac* promoter. Briefly, the PCR-amplified fragments (delta41, delta141, delta217, and delta367) of human POLRMT containing an N-terminal His tag sequence were fused with a PCR-linearized pMal-c5X vector (without a maltose binding protein sequence) using an In-Fusion HD cloning kit (Clontech, Mountain View, CA) and cloned into Stellar cells. A control plasmid carrying POLRMT with a mutation at the catalytically essential active site (D1151A) (17) was constructed by fusion of a chemically synthesized 60-bp DNA fragment containing mutation sites with a PCR-linearized POLRMT expression plasmid (delta141). For protein expression, the plasmids were transformed into Stellar competent cells. The pMal-c5X expression vector contains a *lacI* gene, which allows the inducible expression of POLRMT in Stellar cells. The transformed cells were grown in LB medium containing 100 μg/ml ampicillin at 35°C to an optical density at 600 nm of 1. Cells were cooled to 4°C in a refrigerator for 1 h. MgCl_2_ was added to a final concentration of 1 mM, and protein expression was induced at 16°C overnight by the addition of 0.4 mM IPTG. Cells were harvested by centrifugation at 4,000 × *g* for 20 min at 4°C. The cell pellets were stored at −80°C before further processing.

### Protein purification and identification.

Cell pellets were resuspended in sonication buffer (20 mM Tris-HCl, pH 7.5, 10% glycerol, 500 mM NaCl, 0.5% Triton X-100, 10 mM DTT, 10 mM MgCl_2_, 30 mM imidazole, 1× protease inhibitor cocktail). Cell disruption was performed on ice for 10 min using an ultrasound probe sonicator. The cell extract was clarified by centrifugation at 16,000 × *g* for 20 min at 4°C. The supernatant was incubated with HisPur Ni-NTA agarose resin with gentle rocking for 15 min at 4°C. The resin was then washed 5 times with 10 volumes of wash buffer (20 mM Tris-HCl, pH 7.5, 10% glycerol, 500 mM NaCl, 0.1% Triton X-100, 1 mM DTT, 2 mM MgCl_2_) containing 30 mM imidazole and then once with wash buffer containing 2 M NaCl. The protein was eluted from the resin with 1 volume of elution buffer (20 mM Tris-HCl, pH 7.5, 10% glycerol, 50 mM NaCl, 0.5% Triton X-100, 10 mM DTT, 300 mM imidazole). The glycerol concentration in the eluted enzyme mixture was adjusted to 50%, and the mixture was stored at −80°C before use. Protein identification by mass spectrometry was performed by MS Bioworks (Ann Arbor, MI). The concentration of a targeted protein was measured by SDS-PAGE using BSA (Sigma, St. Louis, MO) as a standard.

### RNA primer and DNA template annealing.

To generate RNA primer/DNA template (P/T) duplexes, 1 μM fluorescently labeled RNA oligonucleotide primer (Cy5.5-RNA) and 10 μM unlabeled DNA template were mixed in 50 mM NaCl-deionized RNase- and DNase-free water, incubated at 95°C for 10 min, and then slowly cooled to room temperature. The annealed P/Ts were stored at −20°C in the dark before use in primer extension assays.

### Native PAGE analysis.

The formation of stable P/T duplexes was tested using native polyacrylamide gel electrophoresis (PAGE). Different annealed P/T duplexes were mixed with glycerol loading buffer (50% glycerol, 90 mM Tris base, 29 mM taurine, 0.1% bromophenol blue) and loaded onto a native 10% polyacrylamide gel (19:1), and electrophoresis was performed at 4°C in 1× TT buffer (90 mM Tris base, 29 mM taurine). After electrophoresis, the gels were scanned using an Odyssey infrared imaging system (LI-COR Biosciences, Lincoln, NE). The images were analyzed using Image Studio software (version 4.0; LI-COR Biosciences, Lincoln, NE).

### Primer extension assay.

A primer extension reaction were performed using a fluorescently labeled RNA primer annealed to a DNA template. The specific P/Ts used in different assays are indicated in each figure. A typical primer extension reaction was performed in a 20-μl reaction mixture containing reaction buffer (5 mM Tris-HCl, pH 7.5, 10 mM DTT, 20 mM MgCl_2_, 0.1% Triton X-100, 0.01 U RNasin, 10% glycerol), 10 nM P/T complex, and 20 nM POLRMT. The reactions were initiated by the addition of rNTPs at a final concentration of 100 μM, unless otherwise specified, followed by incubation for 1 h at 22°C. Different incubation times were used in some experiments, as indicated in the legend to each figure. After incubation, the reaction was quenched by the addition of 20 μl quenching/loading buffer (8 M urea, 90 mM Tris base, 29 mM taurine, 10 mM EDTA, 0.02% SDS, 0.1% bromophenol blue). The quenched samples were denatured at 95°C for 15 min, and the primer extension products were separated using 15% denaturing polyacrylamide gel electrophoresis (urea-PAGE) in 1× TTE buffer (90 mM Tris base, 29 mM taurine, 0.5 mM EDTA). After electrophoresis, the gels were scanned using the Odyssey infrared imaging system. The images were analyzed, and the proper RNA bands were quantified using Image Studio Lite software (version 4.0).

### Analysis of the effect of ribonucleotide analog incorporation on POLRMT RNA synthesis.

The primer extension reactions were performed as described above, and each reaction mixture included three natural rNTPs and the fourth rNTP being replaced by an analog rNTP. Briefly, 10 nM P/T and 20 nM POLRMT were assembled in a reaction buffer (5 mM Tris-HCl, pH 7.5, 10 mM DTT, 20 mM MgCl_2_, 0.1% Triton X-100, 0.01U RNasin, 10% glycerol), and the reaction was initiated by adding both rNTPs and an analog rNTP. The final concentrations of the natural rNTPs and analog 5′-triphosphates were 1 μM and 100 μM, respectively. The reaction mixtures were incubated at 22°C for 1 h, and the reaction was quenched with quenching buffer. The quenched samples were heated at 95°C for 15 min and analyzed by denaturing PAGE as described above.

### Measurement of ribonucleotide analog incorporation efficiency.

Different P/Ts were designed to test individual analog rNTPs using the same principle described previously (18). The particular primers and templates used are shown in each figure. To perform the reaction, 10 nM P/T and 20 nM POLRMT were incubated in reaction buffer (5 mM Tris-HCl, pH 7.5, 10 mM DTT, 20 mM MgCl_2_, 0.1% Triton X-100, 10% glycerol) in the presence of 1 μM the first natural ribonucleotide for 5 min at 22°C, and then different concentrations of the ribonucleotide analogs to be tested were added to the reaction mixtures. The reactions were continued at 22°C for the times indicated in each figure or figure legend and subsequently quenched and analyzed by denaturing PAGE as described above. After electrophoresis, the gels were scanned using the Odyssey infrared imaging system. The intensity of the different RNA bands was quantified using Image Studio Software Lite (version 4.0). The incorporation efficiencies of the different rNTP analogs were evaluated by measurement of the *K*_1/2, analog_ values (the analog triphosphate concentrations resulting in 50% product extension) and the corresponding discrimination values (*D*_analog_; defined as *K*_1/2, analog_/*K*_1/2, natural_, when both were measured under the same assay conditions), as previously described (18).

## Supplementary Material

Supplemental material
