# Supplementary material for: Simple In Vitro Assay To Evaluate the Incorporation Efficiency of Ribonucleotide Analog 5′-Triphosphates into RNA by Human Mitochondrial DNA-Dependent RNA Polymerase
Source: Antimicrob Agents Chemother. 2018 Jan 25;62(2):e01830-17. doi: 10.1128/AAC.01830-17 (PMC5786792; doi:10.1128/AAC.01830-17)
Supplement: Supplemental material [file supp_62_2_e01830-17__index.html]

Supplemental material 

# Simple *In Vitro* Assay To Evaluate the Incorporation Efficiency of Ribonucleotide Analog 5′-Triphosphates into RNA by Human Mitochondrial DNA-Dependent RNA Polymerase

## Supplemental material

- Supplemental file 1 -

  Fig. S1 to S4

  PDF, 1.5M
