# Supplementary material for: Simple In Vitro Assay To Evaluate the Incorporation Efficiency of Ribonucleotide Analog 5′-Triphosphates into RNA by Human Mitochondrial DNA-Dependent RNA Polymerase
Source: Antimicrob Agents Chemother. 2018 Jan 25;62(2):e01830-17. doi: 10.1128/AAC.01830-17 (PMC5786792; doi:10.1128/AAC.01830-17)
Supplement: Supplemental material [file AAC.01830-17_zac002186882s1.pdf]

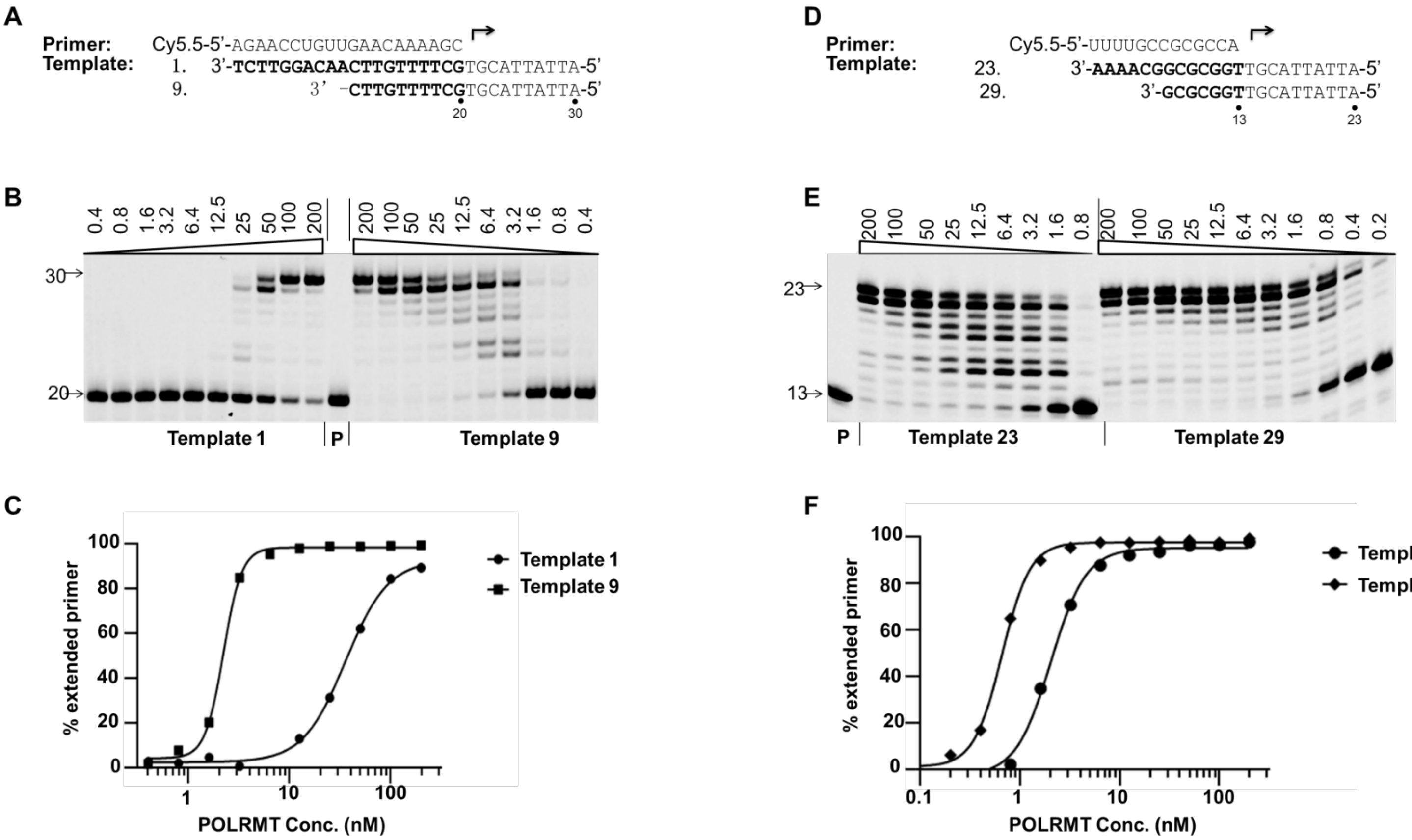

Supplemental Figure S1. Evaluation of the effect of RNA/DNA scaffolds on the efficiency of primer extension reaction catalyzed by POLRMT. (A) RNA primer and DNA templates used in the experiment, and **(B)** a representative gel electrophoresis of the primer extension products. Serial dilutions of POLRMTs, as indicated on the top of the gel (in nM), and a constant concentration of P/T (10 nM) were used in the assay. The template used in each sample is indicated at the bottom of the gel. The reactions were initiated by addition of 100 µM rNTPs; reactions continued at 22 °C for 1 h and then stopped by adding quenching solution. The products were separated on denaturing PAGE. Template used in the reaction is indicated at the bottom of the gel. Number “20” on the left of the gel indicates location of the RNA primer and the “30” indicates location of the extended full-length product. Lane P, no enzyme control. **(C)** The percentages of the full-length products in panel B were plotted against the enzyme concentrations. The results were fitted to sigmoidal dose-response curves using the GraphPad Prism program. The POLTMT concentrations at which half of 20-mer RNA primer is extended into the 30-mer RNA product using template 1 and template 9 are 35.76 nM and 2.217 nM, respectively. **(D)** RNA primer and DNA templates used in the experiment, and **(E)** a representative gel electrophoresis of this primer extension products. The assay was performed similarly as described in panel B. Number “13” on the left of the gel indicates location of the primer used in this assay, and “23” indicates the location of the extended full-length product. **(F)** The percentages of the full-length products in panel E were plotted against the enzyme concentrations using a similar method described in panel C. The POLTMT concentrations at which half of 13-mer RNA primer is extended into the 23-mer RNA product using template 23 and template 29 are 2.029 nM and 0.6565 nM, respectively.

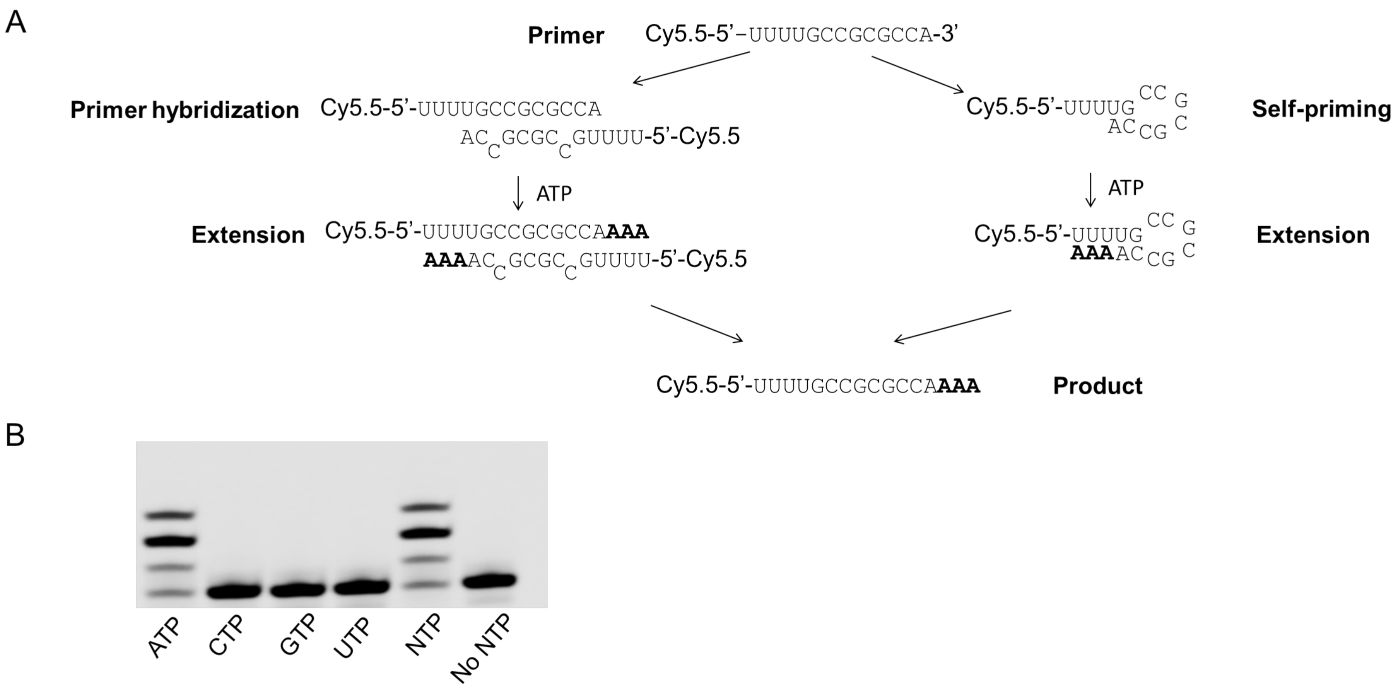

Supplemental Figure S2. Primer extension using RNA as template. (A) Proposed primer-dimerization and self-priming structures, and the primer extension in the presence of ATP. **(B)** POLRMT was incubated with RNA primer from panel A without DNA template. The reactions were initiated by addition of 100 μM different nucleotides (indicated under each lane) and continued for 1 hour at 22 °C. The products were separated on denaturing PAGE. Lane NTP, mixture of ATP, CTP, GTP, UTP.

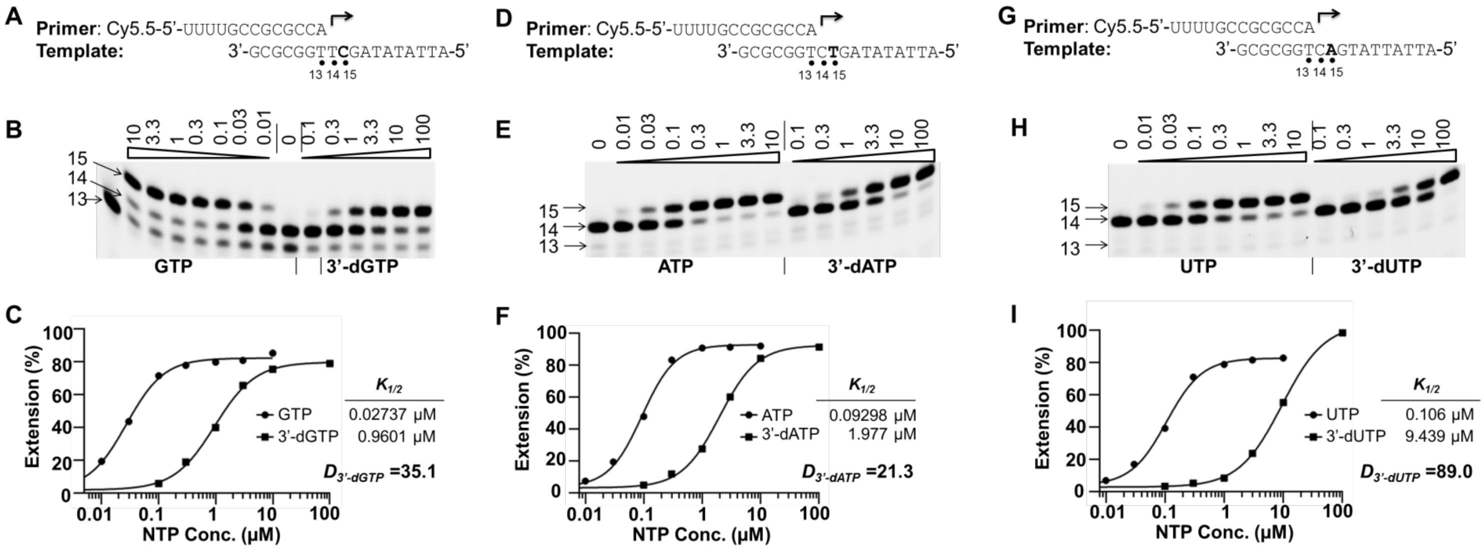

Supplemental Figure S3. Discrimination value measurement of 3'-dGTP, 3'-dATP and 3'-dUTP. (A) Primer and template used to assay GTP analogs. **(B)** A representative image of analysis of $D_{3'\text{-}dGTP}$ values. POLRMT (20 nM) was incubated with 10 nM P/T and 1 μM ATP (the first ribonucleotide to be incorporated) in reaction buffer for 5 min at 22 °C, and then rapidly mixed with different concentrations of 3'-dGTP or GTP, as indicated above each lane (in μM). The reactions were continued at 22 °C for 15 seconds before adding stopping buffer, and the products of the reaction were resolved by denaturing PAGE. The identity of the tested ribonucleotide is indicated at the bottom of the gel. The migrations of the 13-mer primer and 14- and 15-mer first and second ribonucleotide extension products are indicated on the left. **(C)** Quantitative analysis of GTP and 3'-dGTP incorporation in panel B. The incorporation efficiency was evaluated on the basis of the extension of 14-mer to 15-mer products. Discrimination value ($D_{3'\text{-dGTP}}$) was calculated as $K_{1/2,3'\text{-}dGTP}/K_{1/2,\ GTP}$, and is shown on the right of the graph. **(D)** Primer and template used to assay ATP analogs. **(E)** A representative image of analysis of $D_{3'\text{-}dATP}$ values. POLRMT (20 nM) was incubated with 10 nM P/T and 1 μM GTP (the first ribonucleotide to be incorporated) in reaction buffer for 5 min at 22 °C, and then rapidly mixed with different concentrations of 3'-dATP or ATP, as indicated above each lane (in μM). The reactions were continued at 22 °C for 30 seconds before adding stopping buffer, and the products were resolved by denaturing PAGE. **(F)** Quantitative analysis of ATP and 3'-dATP incorporation in panel E. Calculated $D_{3'\text{-dATP}}$ is shown on the right of the graph. **(G)** Primer and template used to assay UTP analogs. **(H)** A representative image of analysis of $D_{3'\text{-}dUTP}$ values. POLRMT (20 nM) was incubated with 10 nM P/T and 1 μM GTP (the first ribonucleotide to be incorporated) in reaction buffer for 5 min at 22 °C, and then rapidly mixed with different concentrations of 3'-dUTP or UTP, as indicated above each lane. The reactions were continued at 22 °C for 60 seconds before adding stopping buffer, and the products were resolved by denaturing PAGE. **(I)** Quantitative analysis of UTP and 3'-dUTP incorporation in the assay in panel H. The calculated $D_{3'\text{-dUTP}}$ is shown on the right of the graph.

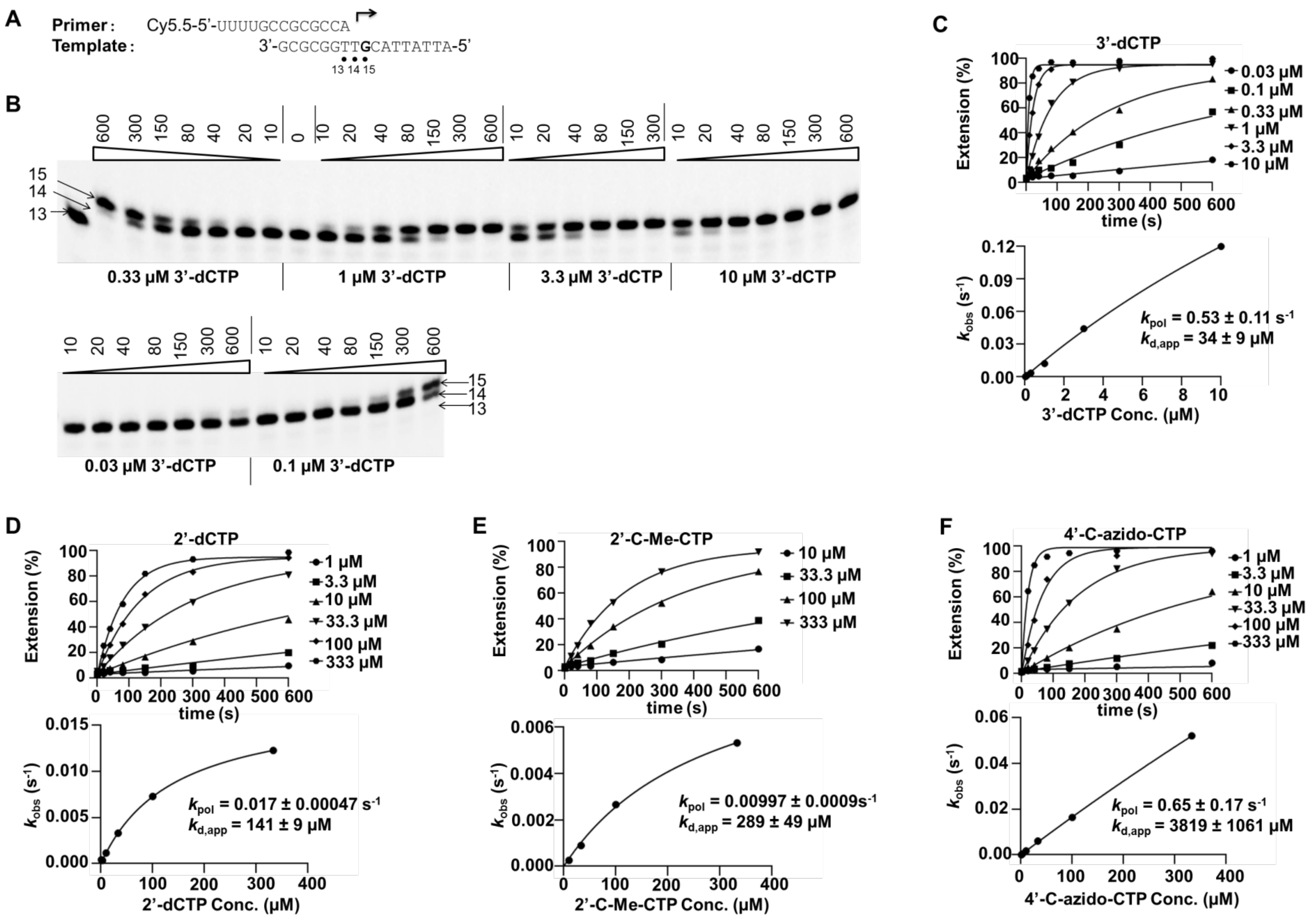

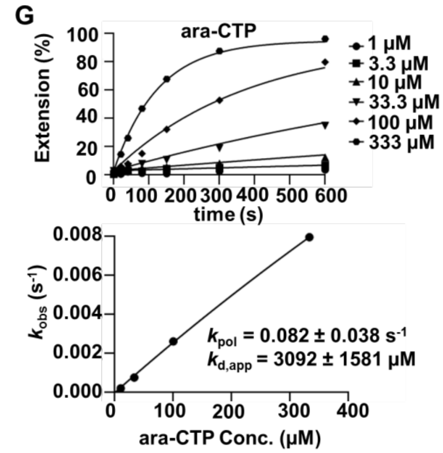

H

Ribonucleotide analog	K_{pol} (s^{-1})	$k_{d,app}$ (μM)	$k_{pol}/k_{d,app}$ ($s^{-1}μM^{-1}$)	D^a	D^*
3'-dCTP	0.53	34	$1.56×10^{-2}$	1	1
2'-dCTP	0.017	141	$1.21×10^{-4}$	128	77.2 ± 3.5
2'-C-Me-CTP	0.00997	289	$3.45×10^{-5}$	452	116 ± 7
ara-CTP	0.082	3092	$2.65×10^{-5}$	589	241 ± 8
4'-C-azido-CTP	0.65	3819	$1.70×10^{-4}$	92	44.9 ± 4.1

Supplemental Figure S4. Pre-steady kinetics of incorporation of CTP analogs. (A) Primer and template used to assay CTP analogs. **(B)** An example of analysis of 3'-dCTP pre-steady state kinetics of incorporation. POLRMT (20 nM) was incubated with 10 nM P/T and 1 μM ATP (the first nucleotide) in reaction buffer for 5 min, then rapidly mixed with different concentrations of 3'-dCTP as indicated at the bottom of the gel. At different time (indicated on the top of the gel) after adding 3'-dCTP, 5 μl aliquots were withdrawn and mixed with 5 μl quenching/loading buffer. The products were analyzed by denaturing PAGE. The locations of the 13-mer primer and 14- and 15-mer first and second ribonucleotide extension products are indicated on the left. **(C)** Top panel is K_{obs} values measurement from (B). The incorporation efficiency was evaluated on the basis of the

extension of 14-mer to 15-mer products. Each time course was fit to a single exponential equation to derive the rate of 3'-dCTP incorporation (K_{obs} values) at each concentration. Bottom panel is K_{pol} and $K_{d,app}$ calculation. Values for K_{obs} were plotted against 3'-dCTP concentrations, and the data was fit to a hyperbola equation to derive the maximum rate of 3'-dCTP incorporation (K_{pol}) and a dissociation equilibrium constant ($K_{d,app}$). The K_{pol} and $K_{d,app}$ values for 3'-dCTP are shown on the the graph. **(D-G)** 2'-dCTP, 2'-C-Me-CTP, 4'-azido-CTP and ara-CTP incorporation and corresponding K_{pol} and $K_{d,app}$ were generated using a similar method as described in panels A-C. **(H)** Summary of several CTP analogs' K_{pol} and $K_{d,app}$ values generated in this assay. The catalytic efficiency ($K_{pol,}/K_{d,app}$) for 3'-dCTP, 2'-dCTP, 2'-C-Me-CTP, ara-CTP and 4'-azido-CTP are 1.56×10^{-2}, 1.21×10^{-4}, 3.45×10^{-5}, 2.65×10^{-5} and 1.70×10^{-4}, respectively. D^{a} is the discrimination value calculated based on the catalytic efficiency of 3'-dCTP and a CTP analog measured in this experiment ($D^{a}_{analog} = (K_{pol,\ 3'\text{-dCTP}}/K_{d,app,\ 3'\text{-dCTP}})/\ (K_{pol,\ analog}/K_{d,app,\ analog})$). D^{*} is discrimination values of the corresponding CTP analogs from **Table 2**.
